# Imagined Examples of Painful Experiences Provided by Chronic Low Back Pain Patients and Attributed a Pain Numerical Rating Score

**DOI:** 10.3389/fnins.2019.01331

**Published:** 2020-02-05

**Authors:** Robert S. Griffin, Maria Antoniak, Phuong Dinh Mac, Vladimir Kramskiy, Seth Waldman, David Mimno

**Affiliations:** ^1^Department of Anesthesiology, Critical Care and Pain Management, Hospital for Special Surgery, New York, NY, United States; ^2^Department of Anesthesiology, Weill Cornell Medical College, New York, NY, United States; ^3^Department of Information Science, Cornell University, Ithaca, NY, United States

**Keywords:** pain numerical rating scale, chronic pain, low back pain, qualitative pain, pain assessment, measurement

## Abstract

**Objective:**

The pain numerical rating scale (NRS) is widely used in pain research and clinical settings to represent pain intensity. For an individual with chronic pain, NRS reporting requires representation of a complex subjective state as a numeral. To evaluate the process of NRS reporting, this study examined the relationship between reported pain NRS levels and imagined painful events reported by study subjects.

**Design:**

A total of 149 subjects with chronic low back pain characterized by the NIH Research Task Force Recommended Minimal Dataset reported current pain NRS and provided imagined examples of painful experiences also attributing to these an NRS. We present a quantitative and qualitative analysis of the 797 pain examples provided by the study subjects.

**Results:**

Study subjects tended to be able to imagine both highly painful 10/10 events and non-painful events with relative agreement across subjects. While NRS for the pain examples tended to increase with example severity, for many types of examples there was wide dispersion around the mean pain level. Examination of pain examples indicated unexpected relationships between current pain and the intensity and nature of the imagined painful events.

**Conclusions:**

Our results indicate that the pain NRS does not provide a reliably interpretable assessment of current physical pain intensity for an individual with chronic pain at a specific moment.

## Introduction

Low back pain has widespread socioeconomic impact worldwide, with an estimated 539,907,000 cases of low back pain in 2015 resulting in its status as the leading cause of years lived with disability globally (Gbd 2015 Diease and Injury Incidence and Prevalence Collaborators, 2016), and has been the subject of extensive research. Both in the back pain literature, and in the clinical care of individuals with low back pain, the problem of evaluating, quantifying, and reporting back pain is a crucial concern (Chapman et al., 2011; Deyo et al., 2014; Chiarotto et al., 2015). The pain numerical rating scale (NRS) is ubiquitously relied upon for the quantification of pain intensity in both research and clinical practice, yet reported as dissatisfactory by pain clinicians (Backonja and Farrar, 2015). The construct validity of the pain NRS, i.e., whether it actually measures what it is used to measure (Elasy and Gaddy, 1998), has not been fully resolved (Jensen et al., 1999; Chapman et al., 2011). This is particularly an issue in chronic pain settings, where the subjective experience of pain and patient’s report of pain may be affected by multiple factors other than sensory pain intensity (Doleys, 2017). From a psychometric standpoint, efforts have been made to evaluate the validity of the pain NRS in terms of its sensitivity to treatments directed to reduce pain intensity, for example (Jensen et al., 1999). Researchers have also attempted to assess criterion validity of the pain NRS with reference to an external standard of painful stimulation in the cold pressor test in a healthy student population (Ferreira-Valente et al., 2011). The extent to which brief cool water immersion is a reasonable “gold standard” for pain intensity experienced by chronic pain patients remains unclear, however.

Several researchers have interrogated the validity of the pain NRS via qualitative inquiry directed at clarifying the process patients undergo as they engage in formulating and providing a pain score. Specifically, de C. Williams and colleagues, in a structured interview approach, found that chronic pain patients reported multiple factors unrelated to sensory pain intensity influence the reported NRS (Williams et al., 2000). In their study, pain patients reported incorporating function and distress into the NRS, as well as influence by social circumstances, while a striking number expressed difficulty with numerically quantifying pain intensity at all. More recently, Robinson-papp et al. (2016) conducted a qualitative focus group study identifying several themes in the attitudes of pain patients to pain NRS reporting: subjects doubted the possibility of measurement of pain as a phenomenon, voiced confusion related to the definition of pain, expressed uncertainty about anchors/referents for the NRS, and expressed difficulty with the concept of “average pain” over a time interval.

Our primary hypothesis was that individuals with chronic low back pain would vary widely in their specific understanding of the pain NRS range, thereby demonstrating that the pain NRS may not be interpretable as a straightforward index of pain intensity level. To test this hypothesis, we asked 149 study subjects with low back pain to report imagined examples of painful events or experiences, and then to attribute a pain NRS to each imagined example. We then conducted a quantitative and qualitative descriptive analysis of the reported pain examples and NRS reports. Eliciting and analyzing pain NRS anchors in an open-ended manner constitutes a novel approach to investigate the pain NRS. We additionally hypothesized, given the discomfort with and uncertainty about interpretation of the NRS score range and anchors reported by Robinson-papp et al. (2016), that study subjects would prefer providing additional qualitatively described experiences as references for their reported pain NRS level to providing the NRS without additional explanatory information.

## Materials and Methods

### Study Design

Institutional review board approval was obtained from the Hospital for Special Surgery. Study subjects were recruited from patients presenting to the Hospital for Special Surgery outpatient pain center for evaluation and management of low back pain and/or lumbar radicular pain between May 12, 2016 and September 1, 2016. Throughout the text, the term “low back pain” will be used to include sciatica and lumbar radicular pain as well as pain localized to the lumbosacral area *per se*. Patients over age 18 years presenting for either new patient evaluation or follow up visit with a primary complaint of either low back pain or lumbar radicular pain were eligible for inclusion. Subjects were excluded if they were unable to speak or write in English, were cognitively impaired, or had 0/10 current back pain on the NRS. Upon enrollment in the study, patients were provided a written survey. The survey included the NIH Pain Consortium Research Task Force (RTF) Recommended Minimum Dataset, the NIH Research Task Force Impact Stratification (Deyo et al., 2014) instrument, and the Pain Catastrophizing Scale (Sullivan et al., 1995). The pain catastrophizing scale is a widely used instrument intended to capture pain-related rumination, magnification, and helplessness (Sullivan et al., 1995). The NIH RTF recommended minimum dataset constitutes a set of key features of medical history, demographics, function, and symptoms, recommended to be reported for all research studies of chronic low back pain (Deyo et al., 2014). The NIH RTF impact stratification instrument, a subset of the recommended minimum dataset, was intended to quantify “personal impact” of low back pain by incorporating self-reported pain intensity, pain interference, and functional status (Deyo et al., 2014) using previously validated items from the PROMIS-29 clinical outcome instrument (Cella et al., 2010; Deyo et al., 2015). T-scores for PROMIS-29 items (Cella et al., 2010) included in the NIH RTF instrument were obtained using www.assessmentcenter.net (PROMIS, RRID:SCR_004718). In addition to the PROMIS-29 (Cella et al., 2010), the NIH RTF recommended minimum dataset includes a two item conjoint substance abuse screen (Brown et al., 2001) and a survey of back pain characteristics and demographics. Subjects were then asked to report current pain numerical rating on a scale of 0–10. Next, subjects were asked to list up to five events or experiences that they felt were less intensely painful than their current pain level, up to five events or experiences that they felt were similar in pain intensity to their current pain intensity level, and up to five events or experiences that they felt were greater in intensity than their current pain intensity level. They were asked to provide examples of pain events that were unrelated to their back pain or sciatica. Subjects were finally asked to report whether they felt that the above information communicated their pain better than, equivalently to, or less well than the pain NRS in isolation. Study data were collected and managed using REDCap electronic data capture tools.

### Data Review and Processing

Qualitative pain examples reported by patients were reviewed by the study investigators, and classified as “abstract” or only painful in the context of a specific painful condition of the study subject. Further analysis was conducted only on examples judged to be abstract. This distinction was made based on whether the reported experience could be interpreted as a painful or otherwise unpleasant experience without the inference of additional patient specific information. For example, “stepped on a nail” or “pain after knee replacement” and “many mosquito bites itching” were considered “abstract” pain-related experiences, while “walking” rated 6/10 was considered specific to the subject’s low back pain or other painful condition. Experiences attributed a NRS of 0/10 or 1/10 were included in further analyses without regard to making a subject specific vs. abstract distinction. For example “walking” was included in additional analysis when rated 0/10 or 1/10, but not when rated 9/10 by the subject. This resulted in a list of subject-reported pain examples attributed a pain NRS by the study subjects. The reported pain examples were then restated by the study investigators to standardize wording while retaining the painful event. For example, “toe stub”, “stubbed toe”, “bumping toe at door frame”, and “stubbing a great toe” were all restated as “stubbed toe”. Next, the restated pain examples were classified according to pain stimulus type (mechanical, thermal, inflammatory, visceral, neuropathic, medical procedure associated, or psychological) and stimulus intensity. Routine daily events without associated physical trauma were classified as “non-painful” or “low intensity,” examples associated with minor trauma were classified as “moderate intensity,” and examples associated with significant potential trauma or injury were classified as “high intensity”.

### Data Analysis

Sample size was selected based on a number expected to be sufficient to support the exploratory, qualitative analysis of a large number of patient examples. Because the analytic approach is novel without a similar study to draw on in the medical literature, sample size calculation could not be empirical. This was felt reasonable given this effort was deemed a preliminary study using a novel analytic approach with no potential harm to the study subjects other than the risks associated with providing survey responses and storing and reviewing that information. The data were primarily displayed in graphical format to facilitate exploratory review of the reported qualitative pain experiences. Univariate association between number of examples and demographic predictor variables was assessed with linear regression for quantitative variables and one-way ANOVA for categorical variables. The Chi square test applied to available cases was used to evaluate preference for reporting pain NRS vs. providing qualitative examples related to pain. Quantitative diagrams demonstrate mean NRS score with error bars indicating 95% confidence intervals calculated from 1000 bootstrap samples from the data. To analyze the association between pain example NRS and pain catastrophizing score, we used a linear mixed effects model with a fixed effect of pain catastrophizing score category and a random intercept by study subject. The likelihood ratio test was used to compare this model to the random intercept model. Data analysis and statistical calculations were done using Python (RRID:SCR_008934) and R (RRID:SCR_001905).

## Results

During the enrollment period of 113 days, 264 potential subjects were approached for consent, and 77 declined to participate. 13 were excluded due to non-English speaking, 12 excluded due to 0/10 current back pain, eleven were excluded due to current injection done at office visit, one was excluded due to cognitive impairment, one was excluded due to age <18, resulting in 149 patients responding to the written interview, comprising 56.4% of those approached for consent. Demographic and back pain characteristics for the study subjects collected according to the NIH Research Task Force recommended minimum dataset for chronic low back pain (Deyo et al., 2015) are presented in Tables 1–4. Study subjects had elevated levels of pain interference and low levels of physical function relative to the United States general population mean based on PROMIS-29 scoring (Table 2). Subjects tended to be low to moderate on the pain catastrophizing scale, with 22.1% of subjects scoring over 30 (Table 2). 65.1% of patients reported “moderate” or “high” pain impact based on the RTF impact classification score (Table 2). 20.8% of subjects were positive on the 2-item conjoint substance abuse screen (Table 1). Study participants tended to have back pain with over 1 years’ duration (69.1%), with back pain either daily or more than half of days (71.8%) (Table 3). Pain at sites other than the low back was frequent in the population. 8.7% of subjects had history of ever having been out of work/unemployed due to back pain for 1 month or more. Opioid use was common in this patient sample, with 47.6% reporting opioid use (Table 4). Among other treatment approaches, 51.7% utilized injections, 69.8% utilized exercise therapy, and 7.4% used psychological counseling (Table 4).

**TABLE 1:** Demographics and comorbid conditions.

Age (years; mean + SD)	57.3 + 15.8
Gender, *N* (% of 149)	
Male	60(40.3%)
Female	89(59.7%)
Race, *N* (% of 149)	
Asian	8(5.4%)
Black/African-American	4(2.7%)
White	133(89.3%)
American Indian/Alaskan Native	2(1.3%)
Unknown/Not reported	3(2.0%)
Decline	0(0%)
Education Level, *N* (% of 149)	
High school or vocational/Occupational	12(8.1%)
Some College	22(14.8%)
Bachelor’s or Associate’s degree	55(36.9%)
Masters’, Professional, or Doctoral	57(38.3%)
Substance abuse, *N* (% of 149)	
Positive two-item conjoint screen	31(20.8%)
Negative two-item conjoint screen	109(73.1%)
Smoking, *N* (% of 149)	
Current or prior smoking	57(38.3%)
No smoking history	88(59.1%)
Obesity class (BMI range), *N* (% of 149)	
Non-obese (18–24.9)	47(31.5%)
Overweight (25–29.9)	62(41.6%)
Class I obesity (30–34.9)	29(19.5%)
Class II obesity (35–39.9)	9(6%)
Class III (Over 40)	1(0.7%)

**TABLE 2:** Pain impact.

**PROMIS29 scores**	**T Score mean + SD**
Pain interference	63.68 + 7.74
Depression/Sadness	50.35 + 9.67
Physical function	36.34 + 6.06
Sleep disturbance	51.22 + 4.46
**Pain catastrophizing scale**	***N* (% of 149)**
Low (less than 20)	66 (44.3)
Moderate (20–29)	43 (28.6)
High (30 and over)	33 (22.1)
**RTF impact classification score**	***N* (% of 149)**
Low (<27)	52 (34.9)
Moderate (28–34)	45 (30.2)
High (>34)	52 (34.9)

**TABLE 3:** Back pain characteristics.

Duration	
Less than 1 month	14(9.4%)
1–6 months	18(12.1%)
6–12 months	13(8.7%)
1–5 years	42(28.2%)
Over 5 years	61(40.9%)
Frequency	
Less than half of days	36(24.8%)
More than half of days	22(14.8%)
Daily	85(57.0%)
Bothered a lot by pain in other areas	
Extremity or joint pain	59(39.6%)
Widespread pain	15(10.1%)
Stomach pain	12(8.1%)
Headaches	11(7.4%)
Prior lumbar spine surgery	
None	111(74.5%)
One prior surgery	24(16.1%)
More than one prior surgery	13(8.7%)
Ever been out of work or unemployed for 1 month or more due to back pain	13(8.7%)
Received disability or compensation due to pain	10(6.7%)

**N* (% of 149).*

**TABLE 4:** Back pain treatment.

**Opioid painkillers**	
Yes – history of use	71(47.6%)
Yes – current use	21(14.1%)
No	69(46.3%)
Not sure	3(2.0%)
No response	6(4.0%)
**Injections**	
Yes	77(51.7%)
No	56(37.5%)
Not sure	1(0.7%)
No response	15(10.1%)
**Exercise therapy**	
Yes	104(69.8%)
No	34(22.8%)
Not sure	1(0.7%)
No response	10(6.7%)
**Psychological counseling**	
Yes	11(7.4%)
No	122(81.9%)
Not sure	1(0.7%)
No response	15(10.1%)

**N* (% of 149).*

**FIGURE 1:**
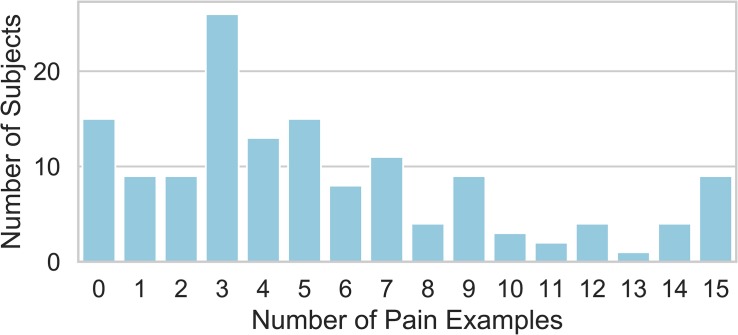
Distribution of the number of pain examples that were attributed a pain NRS by the reporting study subject and could be interpreted without reference to the subject’s specific painful condition and considered for further analysis. Each study subject provided between 0 and 15 total examples.

The study subjects provided a total of 1142 qualitatively reported examples of painful events/states. 83 were provided without an attributed pain NRS and were discarded from further analysis. 10 were associated with an NRS greater than 10, and these were treated as though 10 had been reported consistent with the instructions for the 0–10 pain NRS. Of the remaining pain examples, 262 required a co-existing pain condition to be interpretable as painful and were also discarded from further analysis. This resulted in 797 pain examples, which were studied in the remaining analysis to follow. Study subjects varied in the frequency of pain example provided per patient (Figure 1). There was no univariate association between the number of pain examples for each subject and any of the demographic predictors of age, gender, race, employment, level of education, pain catastrophizing score, substance abuse, BMI, PROMIS-29 subscore, pain duration, pain in other body areas, prior history of surgery, disability status, or prior treatment with opioids, exercise, or psychological therapy (*P* > 0.05 for each).

**FIGURE 2:**
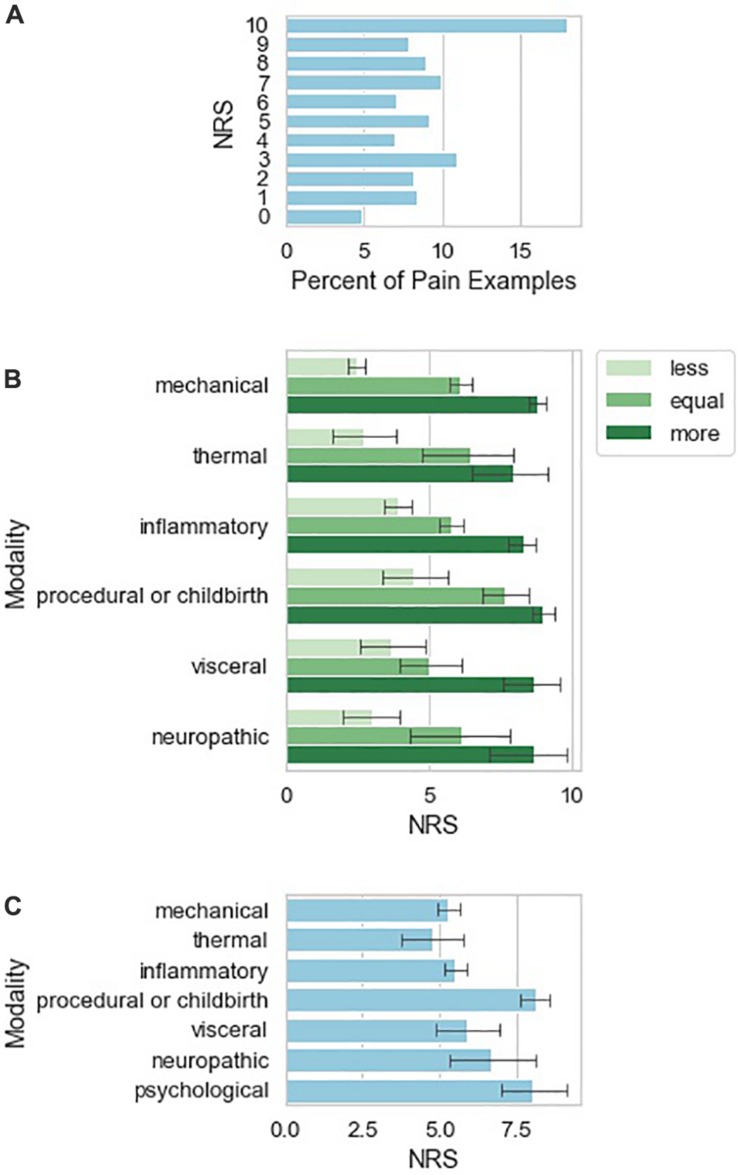
Characteristics of qualitative pain examples provided by study subjects. **(A)** Number of reported pain examples according to pain NRS attributed to the pain example given by the reporting study subject. **(B)** Number of pain examples per investigator-attributed pain classification. Examples are sub-grouped according to whether the study subject indicated the pain example as more painful, similarly painful, or less painful as compared to current low back pain. **(C)** Mean NRS ± 95% confidence interval calculated from 1000 bootstrap samples from the data.

The examples of pain given by patients vary in frequency according to pain NRS score (Figure 2A), with experiences attributed a 10/10 pain score occurring most frequently among the responses. Examples of pain also vary in frequency according to painful stimulus modality (Figure 2B). *Inflammatory* and *mechanical* examples are much more frequent than the other modalities, with *neuropathic* being the least frequent. Figure 2B also shows that examples of *mechanical* modality are more likely to be used as “less painful” while *medical procedures, trauma, or childbirth* are more likely to be used as “more painful” examples. Similarly (Figure 2C), medical procedure associated pain tended to be associated with higher pain NRS than other somatosensory pain types. Two individuals (1.3% of the total study sample) also contributed a total of six pain examples (Table 5) that were purely psychosocial in nature.

**TABLE 5:** Specific examples reported by study subjects (*n* = 2) as painful experiences worse than current pain, which were classified by investigators as “psychological.”

Pain example with attributed NRS	Back pain NRS	PCS	Duration
“Loss of my dog in 2010” 10/10	6 (2–6)	11	Over 5 years

“Deal with solution to family problems” 8/10	6 (6–6)	22	1–5 years
“occurrence of family crises ex: court dates” 8/10			
“loss of very close friend” 7/10			
“disrespect from family members” 7/10			
“family crises like a preemie born in the family” 6/10			

*Examples are reported by reporting study subject, with the pain numerical rating scale (NRS) attributed by the study subject to the event, as well as the subject’s current reported back pain with range best to worst pain in the prior 24 h, the subject’s pain catastrophizing score (PCS), and the subject’s duration of low back pain.*

**FIGURE 3:**
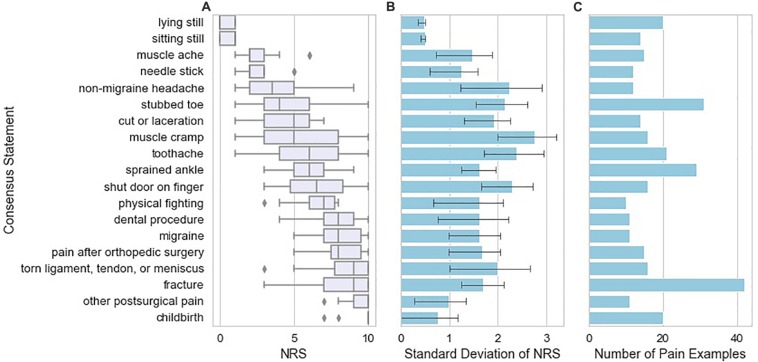
Variation in pain NRS according to the type of example reported by the study subjects. **(A)** NRS attributed by study subjects to the pain examples reported by those subjects, grouped across study subjects as “frequently occurring similar examples” with box plots indicating quartiles of the NRS distribution for each consensus statement. Frequently occurring similar examples reported are those found 10 or more times in the database of pain examples. **(B)** Standard deviation of NRS attributed to the pain example according to the type of pain example. Error bars indicating 95% confidence intervals were calculated from 1000 bootstrap samples from the data. **(C)** Number of pain examples present in the data for each frequently occurring similar example.

Next, we examined the frequency of occurrence and pain score range of pain examples restated from the patient’s exact wording to match patient examples that were highly similar across subjects. We refer to these throughout the manuscript as “frequently occurring similar examples”. The NRS scores for each consensus statement display different levels of variation. Figure 3A shows the quartiles for each of the frequently occurring similar examples that were used as examples at least 10 times by the study subjects. Points on the boxplot indicate the frequency of occurrence of each consensus statement. We observed that certain frequently occurring similar examples were rated consistently across different patients; for example, *childbirth* was almost always rated at an NRS score of 10 among study subjects who reported *childbirth* as an example. On the other hand, some frequently occurring similar examples display high levels of variation: for example, *muscle cramp* varies widely in its NRS score, with a range of 1–10. Figure 3B shows the standard deviation of the NRS scores for each consensus statement, with number shown in Figure 3C. It appears that experiences at either end of the NRS score spectrum tend to be more consistent than experiences falling in the middle of the spectrum.

**FIGURE 4:**
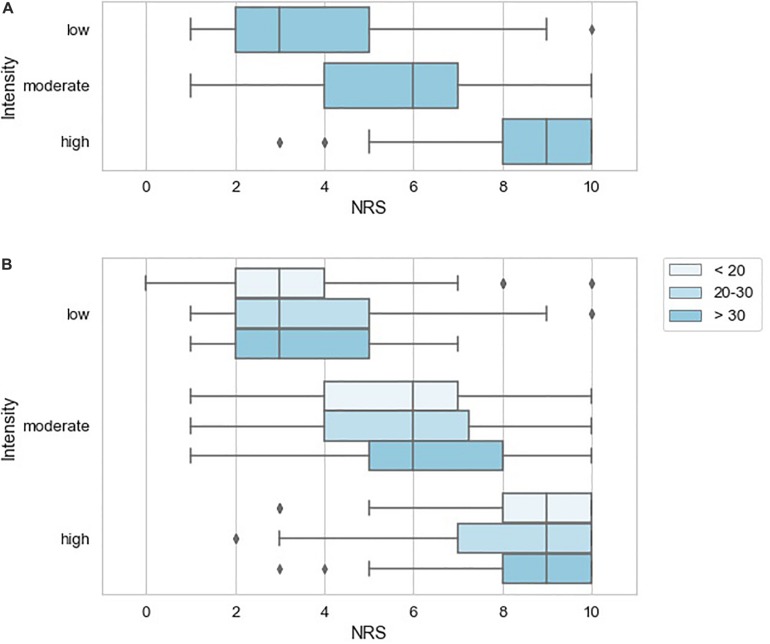
Variation in pain NRS according to stimulus intensity inferred for the reported pain examples. **(A)** NRS according to inferred stimulus intensity with box plots indicating quartiles of the NRS distribution for each pain modality. **(B)** NRS according to inferred stimulus intensity stratified by pain catastrophizing score of the reporting subject with box plots indicating quartiles of the NRS distribution.

To further characterize the pain examples reported by study subjects, we inferred stimulus intensity for each pain example based on the anticipated level of physical trauma that we would expect to be associated with the painful event. Higher-intensity examples tended to be associated with higher NRS scores, but the variation in attributed NRS scores was high, essentially spanning the entire NRS range for low and moderate intensity examples (Figure 4A). We did not observe a tendency for attributed pain NRS examples to vary when stratified by pain catastrophizing score category of the reporting subject (Figure 4B). Similarly, pain catastrophizing score category was not a significant predictor of pain example NRS in a linear mixed effects model either alone or including duration as an additional predictor. To identify instances in which the reported pain NRS associated with a pain example may differ from the expected pain intensity associated with the example given, we prepared a table reporting the specific pain examples of study subjects who reported low intensity stimuli associated with pain NRS of 7 or greater (Table 6; reported by 14/149 subjects), study subjects who reported high-intensity stimuli associated with pain NRS of 2 or less (Table 7A; reported by 2/149 subjects), study subjects who reported unpleasant, non-painful stimuli associated with pain NRS of greater than 2 (Table 7B; reported by 5/149 subjects). These pain examples may alter the interpretation for the subject’s back pain NRS scores. For example, one subject with current 7/10 back pain reported “paper cut 1 h old” as also 7/10 (Table 5), while another subject with current 6/10 back pain reported “burn with curling iron” as 2/10 (Table 7A), and a third subject with current 3/10 back pain reported “many mosquito bites itching” as 4/10.

**TABLE 6:** Specific pain examples provided and attributed NRS ≥ 7 by study subjects (*n* = 14) that were classified as low intensity.

Pain example with attributed NRS	Back pain NRS	PCS	Duration
“Stub toe” 10/10	7 (4–9)	20	Over 5 years

“Walking on hot sand” 10/10	8 (4–8)	23	1–5 years

“Stab a toe” ^∗^ 9/10	7 (3–8)	26	Over 5 years
“earache” 7/10			

“Ear ache” 8/10	10 (1–10)	27	1–3 mos

“Foot being stepped upon” 8/10	3 (1–6)	5	1–5 years

“Stubbing your toe” 8/10	5 (0–9)	13	1–5 years

“Bee sting” 8/10	6 (NA-NA)	29	Over 5 years

“Long bike ride 40 to 100 miles” 8/10	3 (3–5)	14	Over 5 years

“Grit in eye” 8/10	7 (2–9)	21	Over 5 years
“scraped knee” 8/10			
“callus pressing against shoe” 7/10			
“chafed groin” 7/10			
“cramp in hands” 7/10			
“paper cut 1 h old” 7/10			
“twisted arm” 7/10			

“Huge bruise along 5th metatarsal from accidentally kicking steel door frame while barefoot” 7/10	7 (6–8)	16	Over 5 years
“huge poison ivy rash on both legs” 7/10			

“Stub a toe” 7/10	7 (2–8)	30	1 to 5 years

“Stubbing toe short term pain” 7/10	6 (1–7)	13	Over 5 years

“Stepping on broken shells on beach shortlived” 7/10	3 (0–8)	31	1 to 5 years

“Poke with stick” 7/10	7 (4–8)	18	Over 5 years

*Examples are reported according to reporting study subject (row), with the pain numerical rating scale (NRS) attributed by the study subject to the event, as well as the subject’s current reported back pain with range best to worst pain in the prior 24 h, the subject’s pain catastrophizing score (PCS), and the subject’s duration of low back pain. ^∗^This was taken to have meant “stub a toe” rather than “stab a toe.”*

**TABLE 7:** Specific pain examples as reported by study subjects.

A			
**Pain example with attributed NRS**	**Back pain NRS**	**PCS**	**Duration**

“Poke in the eye” 2/10	5 (2–6)	27	Over 5 years

“Burn with curling iron” 2/10	6 (2–8)	20	Over 5 years

**B**			

**Pain**	**Back pain NRS**	**PCS**	**Duration**

“Many mosquito bites itching” 4/10	3 (0–5)	9	Over 5 years
“medium nausea” 4/10			

“Walking for 12 h” 2/10	2 (2–5)	5	Over 5 years

“Mosquito bite” 2/10	3 (1–6)	5	1–5 years

“Standing still straight posture” 2/10	5 (2–7)	24	6–12 mos

“Mosquito bite” 2/10	4 (1–7)	NA	NA

*Examples are reported according to reporting study subject (row), with the pain numerical rating scale (NRS) attributed by the study subject to the event, the subject’s current reported back pain with range best to worst pain in the prior 24 h, the subject’s pain catastrophizing score (PCS), and the subject’s duration of low back pain. **(A)** Examples classified by investigators as “high intensity” associated with subject-attributed NRS ≤ 2 (*n* = 2). **(B)** Examples classified by investigators as “non-painful” associated with subject attributed NRS ≥ 2 (*n* = 5).*

In the total study sample of 149 subjects, who were all seen in the context of an outpatient office visit with no procedure or other medical intervention done at the time of the visit, seven subjects reported current pain of 10/10. Their pain examples and comparison to current pain level are presented (Table 8). 2/7 of these subjects left the section of the questionnaire eliciting pain experiences worse than current pain unanswered, consistent with the expected definition of NRS 10/10 pain. 1/7 of the subjects stated “My answer for number 2 could all be greater,” essentially indicating a variety of disparate pain examples with potentially varying pain NRS relative to one another. 2/7 of the subjects with 10/10 pain modified the NRS 0–10 pain scale by indicating pain examples worse than current pain with numbers greater than 10.

**TABLE 8:** All pain examples reported by study subjects (*n* = 7) with 10/10 current back pain.

Examples where pain is LESS than current pain	Examples where pain is EQUAL to current pain	Examples where pain is GREATER than current pain	Back pain NRS pain catastrophizing (PCS) pain duration
“Headache” 5/10	“Burn” 10/10	“Broken legs” 10/10	10 (6–9)
			PCS 50
			1–5 years

“Cutting finger” 1/10			10 (NA-NA)
			PCS 0
			Over 5 years

“Giving blood” 2/10	“Hitting shin on a bar really hard” 10/10	“Child birth” 14/10	10 (0–10)
“smashing finger on door” 7/10	“walking into a brick wall” 10/10	“cracked tooth” 13/10	PCS 29
“hitting shin on a bar” 8/10		“2nd degree skin burn” 14/10	1–5 years
“getting elbowed in head – hard” 3/10			
“stub toe” 3/10			

“Shoulder pain” 6/10	“Severe toothache” 10/10	“My answer for number 2 could all be greater” ^∗∗∗^	10 (7–10)
“a cut” 6/10	“burn” 10/10		PCS 39
“stubbing my toe” 5/10	“Achilles tendon tear” 10/10		1 to 5 years
	“broken bone” 10/10		
	“recovering from stomach surgeries” 10/10		

“Fractured humerus” 8/10	“Child birth (labor)” 10/10		10 (7–10)
“breast pain due to caffeine” 8/10			PCS 28
			Less than 1 month

“Twisted ankle” 9/10	“Broken bone” 10/10	“Getting shot” 10+/10	10 (1–10)
“shin bruise” 9/10	“migraine” 10/10	“shark bite” 10+/10	PCS 27
“ear ache” 8/10	“muscle tear” 10/10		1 to 3 mos
“toothache” 9/10			

“Paper cut” ^∗^	“Bone bruise”^∗^	“Broken bone”^∗^	10 (2–4)^∗∗^
“blood test”	“workout in gym new leg exercise”	“heart attack”	PCS NA
“stub toe”	“body aches with fever”	“close car door on finger”	Over 5 years back pain
	“migraine”	“car run over foot”	
	“bad leg cramp”	“getting tooth cavity filled no anesthesia”	

*Each row corresponds to an individual study subject. ^∗^Subject provided no pain NRS. ^∗∗^Current NRS with best and worst over last 24 h as reported. ^∗∗∗^Authors infer that in this case “number 2” refers to “Examples where pain is EQUAL to current pain”.*

**FIGURE 5:**
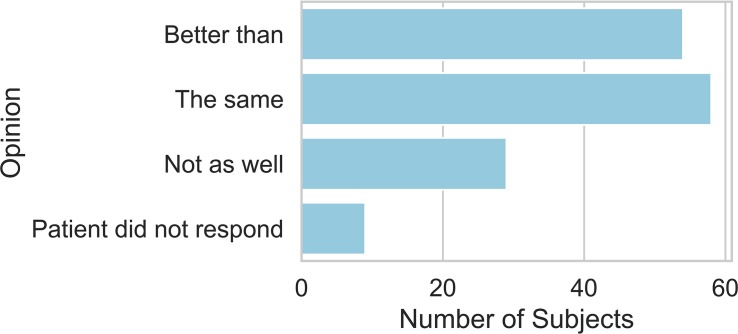
Stated preference for using the NRS alone to report on current pain, no preference, or preference for NRS with pain examples to report on current pain.

Figure 5 shows that 38.6% of patients agreed that the descriptions communicated the intensity of their pain better than the NRS scores. 19.3% of patients thought that the descriptions communicated worse than the NRS scores. This distribution of responses deviated significantly from a uniform distribution (*X*^2^ = 10.5, d.f. = 2, *P* < 0.01).

## Discussion

In this study, we asked study subjects with chronic back pain to use the pain NRS to rate their pain, and to think about or imagine painful states, and then attribute to those imagined examples a pain NRS to help provide context for the process of pain NRS reporting. This was motivated by our interest in the construct validity of the pain NRS in individuals with chronic back pain, particularly in the context of a clinical encounter setting, in which the clinician assumes the task of using the reported pain NRS score to infer the pain intensity state of the patient. We observed a tendency for higher intensity pain examples to be associated with higher pain scores. However, the dispersion observed in our data set was such that outside the extremes of painful or non-painful events, a single pain NRS did not provide reliable information about the intensity of the event the subject was thinking of. We do not expect to unseat the pain NRS as a clinical outcome instrument, given that it is widely used with properties indicating psychometric validity in clinical study settings (Jensen et al., 1999; Farrar et al., 2000, 2001; Chien et al., 2013; Smith et al., 2016). Rather, we question the relentless use of the NRS pain score in clinical settings, in which NRS reports do not benefit from sample-based averaging over many individuals’ idiosyncratic interpretations of the NRS range and anchors, or from the opportunity to train study subjects to improve the accuracy of pain score reporting (Smith et al., 2016; Treister et al., 2018).

There were a number of findings in the study that raise questions about the extent to which any individual pain NRS may be interpretable as a measurement of “pain intensity” or a reliable indicator of a specific individual’s pain state. For pain examples at moderate levels of pain intensity there was wide dispersion in the NRS scores associated with these examples by the subjects reporting them. There was consensus around more extreme pain such as childbirth or postsurgical pain, on the other hand. This latter observation also highlights the context-sensitivity of the pain NRS given that postsurgical pain and labor pain both vary widely between and within individuals, while later memory of these events is likely dependent on the most painful moments (Redelmeier and Kahneman, 1996; Redelmeier et al., 2003; Christensen-Szalanski, 2007). This is consistent with the findings of Ferreira-Valente et al. (2011) in their validation study of the pain NRS using the cold pressor test: while pain NRS was sensitive to changing cold temperature, the standard deviation of pain NRS for each temperature was wide, with both 4/10 and 7/10 within one standard deviation of the mean for each temperature tested. As a result, a 6/10 report in one person would not be useful as a means to infer the probable temperature of the water bath, analogous to the task of using a single pain NRS in a clinical setting to infer an individual’s clinical pain state.

Strikingly, the 10/10 pain score was the most frequently used pain intensity number for the imagined examples provided by the study subjects. Our impression is that the 10/10 pain score indicates communication failure between subject and interviewer or between patient and clinician. Imagined 10/10 pain examples in the current study included stimuli as disparate as “hitting shin on a bar,” “being burned alive,” “childbirth,” “loss of my dog,” “pain after back surgery”, and “plantar fasciitis”. One study participant reporting current 10/10 back pain listed several pain examples as “equal to current pain” including “severe toothache,” “burn,” “Achilles tendon tear,” “broken bone,” and “recovering from stomach surgery,” and for the questionnaire prompt “worse than current pain” simply provided the response “My answer for number 2 could all be greater.” This response highlights the breakdown in communication occurring around the 10/10 anchor point for the pain scale. It is difficult to understand the concept of current 10/10 pain as the worst possible pain in the context of an outpatient ambulatory office visit due to chronic low back pain. The clinician in an office encounter is likely able to imagine various medical circumstances that may seem far more severely painful than what the patient could possibly be experiencing at the moment of the encounter, when the pain NRS is interpreted purely as a measurement of sensory pain intensity level.

While the communication breakdown associated with 10/10 pain NRS was most striking, there were frequent, similar findings with regard to intermediate pain scores in terms of discrepancies between the expected stimulus intensity of an event and the associated NRS score. If an individual describes “paper cut 1 h old” as 7/10 pain NRS should this alter interpretation of that same individual’s report of 7/10 NRS low back pain?

Considering the reported pain NRS in light of Eric Cassel’s framework described in “The Nature of Suffering and the Goals of Medicine” (Cassel, 1982) may suggest that rather than a measurement of pain intensity, the reported NRS may be a reflection of the threat to the individual’s “personhood,” a more complex concept including disruption of self-image and personal plans, as well as altered cultural, familial, and economic roles. This interpretation would help explain the wide range of dispersion in NRS associated with moderately painful events, where the potential threat to person is likely more variable. Similarly, the un-interpretability of individual NRS scores suggested by the present exploratory study is coherent with the hypothetical construct model of pain advocated by Daniel Doelys (Doleys, 2017) in which successful treatment of chronic pain requires analysis and management of a complex system of interacting factors which produce a chronic pain state, rather than excessive preoccupation with sensory pain intensity itself. Such a model calls for an interrogative/narrative based form of pain evaluation (Cepeda et al., 2008; Rosti, 2017) rather than a purely reductionist approach based on the quantitative NRS score. Narrative examples or concrete anchors may not capture the full complexity of painful events which may vary in intensity within a single event, while the memory of pain intensity may differ from contemporaneously reported pain intensity (Redelmeier and Kahneman, 1996; Redelmeier et al., 2003; Daoust et al., 2017), but based on the present study, we suspect these examples will more closely communicate patient’s pain intensity than a purely abstract numeral. More widespread use of multidimensional, comprehensive outcome instruments such as the PROMIS-29 (Cella et al., 2010; Deyo et al., 2015) or more focused instruments oriented toward chronic pain or associated constructs (Turk et al., 2016) such as the CARE Scale-7 (Ziadni et al., 2018), or toward underlying pain mechanisms (Scholz et al., 2009; Vardeh et al., 2016), may be valuable in avoiding some of the false reduction of dimensionality inherent in the use of the NRS in the chronic pain setting.

The present findings underscore the need for improved communication about NRS pain score reporting and interpretation between study investigators and study participants in chronic pain clinical trials, when there is little inter-individual agreement about moderately painful events. Recent research has indicated that chronic pain studies may be improved by pre-training subjects in pain intensity reporting (Smith et al., 2016; Treister et al., 2018). For example, Treister et al. (2018) demonstrated that prior training in pain intensity reporting with reference to a standardized set of mechanically painful stimuli may have potential for reducing placebo effect magnitude in chronic pain studies. Similarly, the action-project study indicated that training study participants in pain intensity reporting may improve NRS discriminant validity (Smith et al., 2016). These observations, given that they indicate that NRS reporting is malleable, further question the utility of raw NRS reports in clinical settings.

The present study has several limitations. First, the study was designed as an exploratory, hypothesis-generating study, and the analysis was primarily qualitative in nature. Second, the study sample was primarily Caucasian and relatively highly educated; this may limit generalizability of the findings. This highlights the potential need for additional qualitative research to investigate attitudes and qualitative responses to NRS scores in subjects with lower levels of education and in samples with greater range of race, ethnicity, and cultural background. Third, the study was limited to chronic low back pain patients, and it is not clear that the present findings would be as relevant to acute pain settings, such as acute postsurgical pain.

## Conclusion

The current exploratory study of qualitative experiences imagined by patients and their association with pain NRS scores indicates a number of potential problems with interpreting pain NRS scores as straightforward measurements of pain intensity level in chronic low back pain patients. Specifically, there is wide dispersion in interpretation of moderately painful events, while the frequent reporting of imagined 10/10 painful events indicates that it may be difficult for individuals to distinguish severely painful events from one another in terms of pain intensity. Going forward, there are a number of potential options that merit additional investigation for revising pain assessment tools. Specifically, it may be valuable to investigate pain scales using concrete examples rather than abstract numerals as anchor points to represent pain intensity. There also may be potential for improved construct validity of pain intensity assessment tools relative to the abstract pain NRS by developing empirical, example-based anchors specific for particular pain contexts, such as pain intensity of chronic low back pain or pain intensity after total knee arthroplasty. The present preliminary study presents data which may be useful as a starting point to support construction of such a scale in the context of chronic low back pain.

## Data Availability Statement

The datasets for this manuscript are not publicly available because they contain protected health information. Requests to access the datasets should be directed to RG, griffinr@hss.edu.

## Ethics Statement

The studies involving human participants were reviewed and approved by the Hospital for Special Surgery Institutional Review Board. The patients/participants provided their written informed consent to participate in this study.

## Author Contributions

RG principally designed the study and participated in data collection, data analysis, and drafted and revised the manuscript. MA conducted the data analysis, prepared the figures, and participated in the revision of the manuscript. PM participated in the study design and data collection. VK and SW participated in data collection and revision of the manuscript. VK participated in the data analysis. DM guided the data analysis plan and participated in drafting and revision of the manuscript.

## Conflict of Interest

The authors declare that the research was conducted in the absence of any commercial or financial relationships that could be construed as a potential conflict of interest.

## References

[B1] Backonja M. Farrar J. T. (2015). Are pain ratings irrelevant? *Pain Med.* 16 1247–1250. 10.1111/pme.12748 26176790

[B2] Brown R. L. Leonard T. Saunders L. A. Papasouliotis O. (2001). A two-item conjoint screen for alcohol and other drug problems. *J. Am. Board Fam. Pract.* 14 95–106. 10.1007/s00339-014-8238-1 11314930

[B3] Cassel E. J. (1982). Cassell_ the-nature-of-suffering-and-the-goals-of-medicine. *N. Engl. J. Med.* 306 639–645. 7057823 10.1056/NEJM198203183061104

[B4] Cella D. Riley W. Stone A. Rothrock N. Reeve B. Yount S. (2010). Initial adult health item banks and first wave testing of the patient-reported outcomes measurement information system (PROMIS TM) network: 2005–2008. *J. Clin. Epidemiol.* 63 1179–1194. 10.1016/j.jclinepi.2010.04.011 20685078 PMC2965562

[B5] Cepeda M. S. Chapman C. R. Miranda N. Sanchez R. Rodriguez C. H. Restrepo A. E. (2008). Emotional disclosure through patient narrative may improve pain and well-being: results of a randomized controlled trial in patients with cancer pain. *J. Pain Symptom Manage.* 35 623–631. 10.1016/j.jpainsymman.2007.08.011 18359604

[B6] Chapman J. R. Norvell D. C. Hermsmeyer J. T. Bransford R. J. Devine J. McGirt M. J. (2011). Evaluating common outcomes for measuring treatment success for chronic low back pain. *Spine* 36 S54–S68. 10.1097/BRS.0b013e31822ef74d 21952190

[B7] Chiarotto A. Deyo R. A. Terwee C. B. Boers M. Buchbinder R. Corbin T. P. (2015). Core outcome domains for clinical trials in non-specific low back pain. *Eur. Spine J.* 24 1127–1142. 10.1007/s00586-015-3892-3 25841358

[B8] Chien C. W. Bagraith K. S. Khan A. Deen M. Strong J. (2013). Comparative responsiveness of verbal and numerical rating scales to measure pain intensity in patients with chronic pain. *J. Pain* 14 1653–1662. 10.1016/j.jpain.2013.08.006 24290445

[B9] Christensen-Szalanski J. J. J. (2007). Discount functions and the measurement of patients’. *Med. Decis. Mak.* 4 47–58. 10.1177/0272989x8400400108 6727587

[B10] Daoust R. Sirois M. J. Lee J. S. Perry J. J. Griffith L. E. Worster A. (2017). Painful memories: reliability of pain intensity recall at 3 months in senior patients. *Pain Res. Manag.* 2017:5983721. 10.1155/2017/5983721 28260963 PMC5312450

[B11] Deyo R. A. Dworkin S. F. Amtmann D. Andersson G. Borenstein D. Carragee E. (2014). Focus article Report of the NIH task force on research standards for chronic low back pain. *Pain Med.* 15 1249–1267. 10.1097/AJP.0000000000000120 25132307

[B12] Deyo R. A. Ramsey K. Buckley D. I. Michaels L. Kobus A. Eckstrom E. (2015). Performance of a patient reported outcomes measurement information system (PROMIS) short form in older adults with chronic musculoskeletal pain. *Pain Med.* 17 314–324. 10.1093/pm/pnv046 26814279 PMC6281027

[B13] Doleys D. M. (2017). Chronic pain as a hypothetical construct: a practical and philosophical consideration. *Front. Psychol.* 8:664. 10.3389/fpsyg.2017.00664 28496426 PMC5406449

[B14] Elasy T. A. Gaddy G. (1998). Measuring subjective outcomes: rethinking reliability and validity. *J. Gen. Intern. Med.* 13 757–761. 10.1046/j.1525-1497.1998.00228.x 9824522 PMC1497034

[B15] Farrar J. T. Portenoy R. K. Berlin J. A. Kinman J. L. Strom B. L. (2000). De^®^ ning the clinically important difference in pain outcome measures. *Pain* 88 287–294. 10.1016/S0304-3959(00)00339-0 11068116

[B16] Farrar J. T. Young J. P. LaMoreaux L. Werth J. L. Poole R. M. (2001). Clinical importance of changes in chronic pain intensity measured on an 11-point numerical pain rating scale. *Pain* 94 149–158. 10.1016/S0304-3959(01)00349-9 11690728

[B17] Ferreira-Valente M. A. Pais-Ribeiro J. L. Jensen M. P. (2011). Validity of four pain intensity rating scales. *Pain* 152 2399–2404. 10.1016/j.pain.2011.07.005 21856077

[B18] Gbd 2015 Diease and Injury Incidence and Prevalence Collaborators. (2016). Global, regional, and national incidence, prevalence, and years lived with disability for 310 diseases and injuries, 1990–2015: a systematic analysis for the Global Burden of Disease Study 2015. *Lancet* 388 1545–1602. 10.1016/S0140-6736(16)31678-6 27733282 PMC5055577

[B19] Jensen M. P. Turner J. A. Romono J. M. Fisher L. D. (1999). Comparative reliability and valididty of chronic pain intensity measures. *Pain* 83 157–162. 10.1016/s0304-3959(99)00101-3 10534586

[B20] Redelmeier D. A. Kahneman D. (1996). Patients’ memories of painful medical treatments: real-time and retrospective evaluations of two minimally invasive procedures. *Pain* 66 3–8. 10.1109/PTC.2015.7232658 8857625

[B21] Redelmeier D. A. Katz J. Kahneman D. (2003). Memories of colonoscopy: a randomized trial. *Pain* 104 187–194. 10.1016/S0304-3959(03)00003-4 12855328

[B22] Robinson-papp J. George M. C. Dorfman D. Simpson D. M. (2016). Barriers to chronic pain measurement: a qualitative study of patient perspectives. *Pain Med.* 16 1256–1264. 10.1111/pme.12717.Barriers 25688752 PMC4504818

[B23] Rosti G. (2017). Role of narrative-based medicine in proper patient assessment. *Support. Care Cancer* 25 3–6. 10.1007/s00520-017-3637-4 28220317 PMC5357296

[B24] Scholz J. Mannion R. J. Hord D. E. Griffin R. S. Rawal B. Zheng H. (2009). A novel tool for the assessment of pain: validation in low back pain. *PLoS Med.* 6:e1000047. 10.1371/journal.pmed.1000047 19360087 PMC2661253

[B25] Smith S. M. Amtmann D. Askew R. L. Gewandter J. S. Hunsinger M. Jensen M. P. (2016). Pain intensity rating training: results from an exploratory study of the ACTTION PROTECCT system. *Pain* 157 1056–1064. 10.1097/j.pain.0000000000000502 27058680

[B26] Sullivan M. J. L. Bishop S. R. Pivik J. (1995). The pain catastrophizing scale: development and validation. *Psychol. Assess.* 7 524–532. 10.1037/1040-3590.7.4.524

[B27] Treister R. Lawal O. D. Shecter J. D. Khurana N. Bothmer J. Field M. (2018). Accurate pain reporting training diminishes the placebo response: results from a randomised, double-blind, crossover trial. *PLoS One* 13:e0197844. 10.1371/journal.pone.0197844 29795665 PMC5993117

[B28] Turk D. C. Fillingim R. B. Ohrbach R. Patel K. V. (2016). Assessment of psychosocial and functional impact of chronic pain. *J. Pain* 17 T21–T49. 10.1016/j.jpain.2016.02.006 27586830

[B29] Vardeh D. Mannion R. J. Woolf C. J. (2016). Towards a mechanism-based approach to pain diagnosis HHS public access. *J. Pain* 17 50–69. 10.1016/j.jpain.2016.03.001 27586831 PMC5012312

[B30] Williams A. C. D. C. Davies H. T. O. Chadury Y. (2000). Simple pain rating scales hide complex idiosyncratic meanings. *Pain* 85 457–463. 10.1016/S0304-3959(99)00299-7 10781919

[B31] Ziadni M. You D. S. Wilson A. C. Darnall B. D. You D. S. (2018). CARE Scale-7: development and preliminary validation of a measure to assess factors impacting self-care in chronic pain. *Clin. J. Pain* 34 818–824. 10.1097/AJP.0000000000000606 29554031 PMC6070413

